# EJNMMI Radiopharmacy and Chemistry obtains its first impact factor!!

**DOI:** 10.1186/s41181-023-00201-7

**Published:** 2023-07-25

**Authors:** Philip H. Elsinga

**Affiliations:** University Medical Center Groningen, Groningen, The Netherlands

EJNMMI Radiopharmacy and Chemistry has obtained its first Impact factor by Clarivate. The journal is now fully indexed and listed in all relevant databases, a list of the indexing services where the journal is included is available at https://ejnmmipharmchem.springeropen.com/about.

The Impact Factor 2022 of EJNMMI Radiopharmacy and Chemistry is 4.6. The journal is included in the following JCR categories:

- CHEMISTRY, MEDICINAL - ESCI.
- RADIOLOGY, NUCLEAR MEDICINE & MEDICAL IMAGING - ESCI.
- CHEMISTRY, INORGANIC & NUCLEAR - ESCI.

The journal has published impactful research and review articles which resulted in this very encouraging Impact Factor which proves the importance of the journal in the field of radiopharmacy and radiochemistry.

Thank you very much to all authors for their submissions as well as to the reviewers for evaluating the papers. Also a big thank to the Editors and Editorial Board Members for their ongoing support, input and ideas and for reviewing. The Editorial Board consists now of 80 scientists in the field and is continously expanding. Recently new Board members from Africa and South America were introduced aiming to strengthen our field in these continents. New Reviewers and Board Members are always welcome.

EJNMMI RPC covers the content of new research in the field of development of new imaging and radionuclide-based therapeutic agents for application in nuclear medicine and molecular imaging. The journal provides a platform for chemists, pharmacists and basic scientists to present their views and scientific work. In addition, the journal provides insight into novel concepts of imaging or radionuclide-based therapeutic agent applications of relevance for the whole molecular imaging community.

Based on recent developments in the field and additional needs of the readership the journal is now also interested in production methods for radionuclides, emerging radionuclide therapy, Artificial Intelligence is emerging and found its application in the Radiopharmaceutical field, the need for harmonization of methods (Best practice), and guidance (guidelines, position papers), abstracts and proceedings of congresses.

A collection is open for submission dedicated only to Best Practices in order to support the field in implementing novel methodologies and attempting to achieve more harmonization of methods.

I hope to continue all the good work and aim that EJNMMI Radiopharmacy and Chemistry can further increase and establish its role being a strong platform for communication and publications in the field of Radiopharmacy and Radiochemistry.

I am very much looking forward to receive your submissions to EJNMMI Radiopharmacy and Chemistry. The online submission and the submission guidelines for the considered article types are available on the journal website https://ejnmmipharmchem.springeropen.com/ where also additional metrics like downloads and turnaround times are listed.

Thank you for your ongoing support.

Philip Elsinga, Editor-in-Chief.

